# Construction and content validation of a checklist for effective communication of nursing care to newborns

**DOI:** 10.1590/0034-7167-2025-0407

**Published:** 2026-09-21

**Authors:** Mychelangela de Assis Brito, Cristianne Teixeira Carneiro, Ruth Cardoso Rocha, José Wicto Pereira Borges, Maria Augusta Rocha Bezerra, Silvana Santiago da Rocha

**Affiliations:** I Universidade Federal do Piauí. Floriano, Piauí, Brazil; II Universidade Federal do Piauí. Teresina, Piauí, Brazil

**Keywords:** Infant, Newborn, Patient Safety, Communication, Checklist, Validation Study., Recién Nacido, Seguridad del Paciente, Comunicación, Lista de Verificación, Estudio de Validación.

## Abstract

**Objectives:**

to construct and validate the content of a checklist to support communication of nursing care for newborns admitted to a neonatal intensive care unit.

**Methods:**

a methodological study conducted in two stages. The first involved three dimensions: theoretical, grounded in an integrative literature review; operational, with the development of the initial version of the checklist; and empirical, carried out through the “Plan, Do, Check, Act” cycle with the participation of 15 nurses from a neonatal intensive care unit. The second stage consisted of content validation by 19 experts, using the Content Validity Ratio.

**Results:**

the final checklist consisted of 84 items, distributed as follows: 31 items in the Procedures category, 23 in Checking, and 30 in Documentation. The overall Content Validity Ratio reached an average of 0.948.

**Conclusions:**

the checklist was validated in terms of content by consensus of the expert committee and can be used in clinical practice.

## RESUMEN

**Objetivos:**

construir y validar el contenido de una lista de verificación para apoyar la comunicación de los cuidados de enfermería a los recién nacidos internados en una unidad de cuidados intensivos neonatales.

**Métodos:**

estudio metodológico desarrollado en dos etapas. La primera involucró tres dimensiones: teórica, fundamentada en una revisión integradora de la literatura; operativa, con la elaboración de la versión inicial de la lista de verificación; y empírica, realizada mediante el ciclo “Planificar, Hacer, Verificar, Actuar”, con la participación de 15 enfermeras de una unidad de cuidados intensivos neonatales. La segunda etapa consistió en la validación de contenido por 19 especialistas, utilizando la Razón de Validez de Contenido.

**Resultados:**

la lista de verificación final estuvo compuesta por 84 ítems, distribuidos en: 31 ítems en la categoría Procedimientos, 23 en Verificación y 30 en Registros. La Razón de Validez de Contenido alcanzó un promedio global de 0,948.

**Conclusiones:**

la lista de verificación fue validada en cuanto a su contenido por consenso del comité de especialistas y puede ser utilizada en la práctica asistencial.

## INTRODUCTION

The Neonatal Intensive Care Unit (NICU) is a highly complex therapeutic environment designed for the care of newborns (NB) at risk^(1)^, but it presents significant susceptibility to the occurrence of adverse events (AEs) due to the coexistence of multiple structural and procedural threats^(2)^. Among these AEs, medication errors, equipment failures, diagnostic inaccuracies, communication problems among professionals, documentation failures, and deficiencies in hygiene practices stand out^(3)^.

Approximately half of the newborns admitted to NICUs experience at least one AE^(4)^, most of which can be classified as preventable or probably preventable^(3)^. Such events are among the leading causes of injury and mortality in neonatal patients^(5)^, highlighting the need to develop and implement preventive strategies aimed at reducing their occurrence and thus promoting patient safety^(6)^.

The adoption of changes in clinical practice, as well as the identification and control of individual and systemic risk factors, is essential to minimize AEs in NICUs^(7)^. In this context, communication stands out as a fundamental pillar and is recognized as one of the main determinants in promoting patient safety^(8)^.

Effective communication is crucial in hospital settings, where different health professionals work in an integrated manner. Nurses, in particular, play a central role in this process, as they interact constantly with various professional categories^(9)^. Satisfaction with communication also directly influences the quality of care provided^(8)^, and numerous studies have shown that failures in interpersonal and interprofessional communication are responsible for a large proportion of preventable AEs in healthcare services^(10,11)^.

There is a clear relationship between effective communication, teamwork, and performance in care safety^(12)^, with structured communication procedures capable of reducing preventable AEs by up to 30% of cases^(13)^. Effective communication encompasses strategies to ensure the clear and accurate transmission of information, contributing to risk reduction and increased safety for both patients and the professionals involved in care^(14)^. Conversely, informational gaps during shift handovers, the absence of standardized protocols, multitasking, distractions, and time pressure are identified as factors that compromise communication quality^(11)^.

Recent studies indicate that the use of standardized scripts, such as checklists - especially during interprofessional rounds - has a positive impact on clinical outcomes^(11,15)^. In the specific context of nursing, the use of such scripts improves communication, reduces the occurrence of adverse events, and promotes safe, high quality care across different healthcare settings^(16)^. In addition to standardizing care and reducing variability in information transmission, checklists enhance teamwork effectiveness and communication among healthcare professionals^(17)^.

Although there have been advances in the use of checklists in critical care environments, studies focusing specifically on the applicability of these instruments in NICUs remain scarce^(16,18)^. There is also a recognized need for ongoing research aimed at adapting checklists to the particularities of neonatal units and the specific needs of newborns^(17)^. Internationally, several nursing checklists have been developed to promote the safety of care provided to newborns hospitalized in NICUs^(2,16)^, as well as in Brazil^(6,18)^. However, to date, no validated checklists have been identified for nurses working in NICU newborn care that specifically standardize the different aspects of communication during shift handovers.

## Relevance of the study

This article is derived from the thesis^(19)^ that is stored at the institutional depository http://repositorio.ufpi.br:8080/xmlui/handle/123456789/324.

## OBJECTIVES

To construct and validate the content of a checklist to support communication of nursing care for newborns admitted to the NICU.

## METHODS

### Study design

This is a methodological study conducted in two stages: construction of the checklist and content validation. Both stages are grounded in the principles of psychometrics, which guide the development of health measurement instruments, particularly in the operationalization of the construct through items^(20)^. The research follows the Standards for Quality Improvement Reporting Excellence (SQUIRE 2.0) guidelines, as it represents an approach aimed at improving healthcare delivery.

### Study procedures

The stages of the study were structured with specific procedures for implementation and data collection. Stage 1, corresponding to the construction of the checklist, was subdivided into three interdependent phases: (1) theoretical dimension, (2) operational dimension, and (3) empirical dimension.

In the theoretical dimension, an integrative literature review (ILR)^(21)^ on effective communication in healthcare was conducted with the aim of identifying relevant empirical indicators for the development of the checklist items. The research question was structured using the PICo strategy - P (patient safety), I (effective communication), Co (neonatology) - formulated as follows: “What strategies are used to ensure effective communication among healthcare professionals to promote the safety of newborns hospitalized in the NICU?”. The search was carried out through the CAPES Portal, accessed via the *Universidade Federal do Piauí* (UFPI, Federal University of Piauí), covering six databases.

In the operational dimension, the first version of the checklist was developed based on the ILR findings and the specificities of care practices in neonatal units. The development of the checklist followed the guidelines of the World Health Organization’s International Patient Safety Goals and the Brazilian National Patient Safety Program^(22,23)^. The intended use and scope of the checklist were defined. Subsequently, descriptors were selected and items were organized into categories with clear conceptual definitions. The wording of the items was objective and direct, facilitating quick responses from the nursing team.

The empirical dimension aimed to assess the applicability of the initial version of the checklists to the care setting. For this purpose, the PDCA cycle methodology (Plan, Do, Check, Act) was adopted, as it is recognized for its effectiveness in promoting continuous improvement in healthcare quality and safety^(24)^. Nurses from the NICU of the regional hospital participated in the development of the second version of the checklist, following the presentation of the first version by the research team. Focus groups were conducted with the participants to integrate scientific knowledge with practical experience. This phase allowed for the inclusion, exclusion, and modification of items, resulting in the consolidation of the second version of the checklist. At this stage, a participant characterization questionnaire was also administered.

In Stage 2, corresponding to the content validation of the checklist, data collection was carried out virtually using a Google Forms^®^ questionnaire sent via email and WhatsApp Messenger^®^. The link included an invitation letter, a consent request (with the Informed Consent Form - ICF), and the forms for participant characterization and checklist item evaluation. A 15 day deadline was established for returning the materials, in accordance with the study timeline.

### Data collection location

Phases 1 (theoretical dimension) and 2 (operational dimension) of the checklist construction (Stage 1) were carried out in the teaching laboratories of the Federal University of Piauí (UFPI), on an inland campus, with the participation of faculty members from the Bachelor of Nursing program. The third phase of this stage, the empirical dimension, took place in a private room of a regional hospital in the state of Piauí. The content validation of the checklist (Stage 2) was conducted in a virtual environment using the Lattes Platform^®^, Google Drive^®^, and Google Forms^®^.

### Period

The study was carried out from the month of March 2020 to August 2021

### Population

For the operational phase of Stage 1, the population consisted of all 27 nurses working in the neonatal care areas of the regional hospital: six assigned to the Neonatal Unit, 15 to the Rooming In Unit, and six to the NICU. In Stage 2, the experts were recruited from across Brazil through the Lattes Platform^®^, which openly compiles researchers’ curricula (Lattes Curriculum) as well as information on research groups and institutions in the country.

### Selection criteria

In Stage 1, for the construction and operationalization of the checklist, the specialists were selected based on the following eligibility criteria: working in the Neonatal Unit, Rooming In Unit, and/or Neonatal Intensive Care Unit (NICU), and having experience in the NICU. Twelve participants were excluded for the following reasons: one nurse was on vacation, two were on medical leave, two did not respond to the invitation to participate in the meetings, two did not attend all meetings, two did not return the instruments after completing the cycle, and three declined to participate, stating they had experience only in obstetrics.

In Stage 2, the construct validation, the specialists were recruited in two ways. Initially, a search was conducted on the Lattes Platform^®^ using the terms “neonatology”, “technologies”, “health technologies”, “nursing technologies”, “patient safety”, and “validation studies” in the “Subject” field. Filters applied included academic background (all), field of activity (health sciences), and language (Portuguese). The candidates’ curricula were analyzed regarding practical and academic experience, participation in research projects, relevant scientific production, and involvement in the topics of interest. The selection of specialists followed criteria that prioritize clinical experience in validation studies in the field of nursing^(25)^.

Specialists who responded to the data collection instrument were asked to indicate two researchers for participation in the material validation through the snowball sampling technique, a method suitable for identifying professionals with highly specific profiles who are difficult to locate through conventional methods^(26)^.

The inclusion criteria for specialists were: being a healthcare professional, working in Brazil, and achieving at least six points in the Criteria for Selecting Experts in the Field^(25)^, considering: at least four years of experience in the specific area (four points), at least one year of teaching experience in the specific area (three points), research experience with articles published in reference journals in the specific area (two points), participation for at least two years in a research group in the specific area (two points), a doctoral degree in the specific area (two points), a master’s degree in the specific area (one point), and residency and/or specialization in the specific area (one point). The specific areas were defined as “health and/or nursing technologies, neonatology, patient safety, and validation studies”. As an exclusion criterion, individuals whose native language was not Brazilian Portuguese were excluded, as this is the language of the checklist.

### Sample definition

In Stage 1, the convenience sample consisted of 15 nurses. In Stage 2, the sample of specialists was defined considering a minimum of six and a maximum of 20 experts^(20)^, with 19 participants contributing.

### Study variables

For the characterization of the nurses, the following variables were collected: sex, age, educational level, academic degree, length of professional experience and time working in neonatology, work unit, and training in neonatology and patient safety. For the specialists, the variables considered were sex, age, geographic origin, education, academic degree, and experience in the study areas. Regarding the validation of the checklist, the psychometric attributes of objectivity, clarity, simplicity, and relevance were evaluated.

### Tools used for data collection

In Stage 1, two instruments were applied: a sociodemographic questionnaire and an analysis guide for the first version of the checklist, with fields for suggestions. In Stage 2, two instruments were used: a sociodemographic questionnaire and an item evaluation form for the checklist, based on four of the 12 psychometric attributes: Objectivity, Clarity, Simplicity, and Relevance^(20)^. Responses were classified as: 1 = yes, 2 = no, 0 = unable to judge.

### Treatment and data analysis

The data were organized in Excel for Windows (version 2110). Frequencies were used for categorical variables, and descriptive measures (mean, minimum, maximum, standard deviation) were used for continuous variables.

Item validation employed the Content Validity Ratio (CVR), using the formula: CVR = (ne - N/2) / (N/2), where *ne* is the number of specialists who rated the item as essential and N is the total number of specialists^(27)^. Items with CVR I ≥ 0.737 were considered valid. Items scoring below this threshold in “Relevance” were excluded; the remaining items were revised based on expert suggestions and submitted to a second validation round. Additionally, the Concordance Index was adopted, with a cutoff point of 0.80^(20)^.

In the second round, a reduced group (5 specialists) was used to reassess the adjusted items^(28)^. Specialists who had provided relevant qualitative contributions were selected. The cutoff point adopted was CVR = 1.000^(27)^.

### Ethical aspects

The study was conducted in accordance with national and international ethical guidelines and was approved by the Research Ethics Committee of the Federal University of Piauí. Informed consent was obtained from the specialists in both data collection phases, in an online format. The approval statement is attached to this submission. Physical documents were stored in a locked cabinet in the office of the principal investigator, while digital files were kept in password protected folders on her computer, with access restricted exclusively to the research team. In all stages of the study, participant anonymity was ensured, both in face to face activities and in those conducted virtually.

## RESULTS

### First stage: Building the *checklist*

A total of 15 nurses participated in this stage. The profile of the participants showed that all were female (100%), aged between 27 and 41 years (mean of 33.5 ± 4.5 years). The length of professional training ranged from two to 12 years, with a mean of 7.7 ± 3.0 years. Regarding academic qualifications, 13 (86.7%) held a *Lato Sensu* postgraduate degree, and only two (13.3%) had an undergraduate degree as their highest qualification. The mean time working in the neonatal area was 2.8 ± 2.1 years. Concerning work units, seven nurses (46.7%) worked in the rooming in unit, three (20%) in neonatology, and five (33.3%) in the NICU. Only four nurses (26.7%) reported having a specialization in Neonatology, and four (26.7%) indicated specific training in patient safety.

The construction of the first version of the checklist on effective communication among nursing professionals aimed at patient safety in the NICU followed clear guidelines for visual and content organization. The checklist was formatted in Times New Roman font, black color, size 12, distributed across two pages with a gray tone layout. Structurally, the checklist was divided into two sections: the first, titled “Purpose of the checklist and completion guidelines”, and the second, composed of the items to be assessed. This second section was arranged in two columns: “Items to be checked” and “Responses”, with the response options: Yes, No, and Not applicable (N/A). The items were grouped into three main categories: Notes (9 items), Checking (17 items), and Procedures (15 items), totaling 41 items.

The revised version of the checklist resulted in a total of 60 items, redistributed into the categories Procedures (22 items), Checking (21 items), and Notes (16 items). All items suggested by the participants were incorporated, considering their relevance to care practice and the specific context of the NICU. The second version of the checklist, now more comprehensive and aligned with clinical reality, was submitted to the next stage of content validation.

### Second stage: validation of the *checklist*

The content analysis of the checklist was conducted in two successive rounds, as recommended in methodological studies focused on instrument validation in the health field. In the first round, 19 Brazilian specialists participated, aged between 25 and 68 years (mean of 37.7 ± 9.2 years), most of whom were female (n = 15; 78.9%). Participants represented four of the five Brazilian regions (North, Northeast, South, and Southeast), predominantly the Northeast (n = 16; 84.2%), and seven states (RR, PI, MA, CE, PE, PR, SP), with the state of Piauí accounting for the largest share (n = 11; 57.9%). Most participants held a degree in Nursing (n = 16; 84.2%) and had between three and 45 years of professional experience (mean of 13.5 ± 8.9 years). Notably, graduates in Medicine (n = 2; 10.05%) and Physiotherapy (n = 1; 5.3%) also participated. Regarding academic qualifications, 10 specialists (52.6%) held a master’s degree in areas related to the study theme, such as neonatology, patient safety, health and nursing technologies, and validation studies.

The evaluation instrument sent to the specialists was structured to allow comments and suggestions across different dimensions of the checklist: title, purpose and completion guidelines, organization by categories (Procedures, Checking, and Notes), and layout. Regarding the title, 4 specialists (21.05%) provided observations. For the completion guidelines, contributions were recorded from 3 evaluators (15.78%). The categorical structure of the checklist received suggestions from 4 specialists (21.05%), while layout related aspects were commented on by only 2 participants (10.52%).

With respect to the CVR of the items in the “Procedures” category, the results obtained - both individual (CVR I) and scale level (CVR S) - were within the agreement parameters established in the literature, as shown in Table 1. However, three items presented individual agreement indices below the critical value (0.737): “drains/catheters/tubes performed” and “transfer performed” for the attribute Clarity, and “newborn’s bath performed” for the attribute Relevance, each with a CVR I of 0.684. These items were modified according to the specialists’ suggestions and forwarded for reassessment in the second round.

**Table 1:** Concordance Index (objectivity, clarity, simplicity, and relevance) and Content Validity Ratio of the “Procedures” section of the checklist by attributes and overall, according to the evaluation of the specialists (N=19), Piauí, Brazil, 2021

Procedures	Objectivity	Clarity	Simplicity	Relevance	CVR-I
Physical examination performed	1.000	1.000	0.894	1.000	**0.974**
Vital signs checked	0.894	0.894	0.789	0.894	**0.868**
Intravenous hydration performed	0.894	0.789	0.894	1.000	**0.894**
Fluid balance performed	1.000	1.000	1.000	1.000	**1.000**
Medications administered	0.894	0.789	0.789	1.000	**0.868**
Diet administered	1.000	0.894	0.789	1.000	**0.921**
Laboratory tests performed	1.000	0.894	1.000	1.000	**0.974**
Imaging exams (X-ray, CT, Echo, Ultrasound) performed	1.000	0.894	1.000	1.000	**0.974**
Surgical procedures performed	0.894	1.000	1.000	1.000	**0.974**
Newborn’s bath performed	0.894	0.894	0.789	0.684	**0.815**
Dressings performed	0.894	0.894	1.000	0.894	**0.921**
Drains/catheters/tubes managed	0.789	0.684	1.000	0.894	**0.842**
Drain, catheter, and tube change/cleaning performed	1.000	0.894	0.894	1.000	**0.947**
Peripheral IV insertion/change performed	1.000	0.894	0.789	1.000	**0.921**
PICC insertion/change performed	1.000	0.894	0.894	1.000	**0.947**
Insertion/change/cleaning of invasive and non-invasive ventilation interfaces performed	1.000	0.789	0.894	1.000	**0.921**
Adoption of fall-prevention measures	1.000	0.789	1.000	1.000	**0.947**
Adoption of pressure-injury prevention measures	1.000	0.789	0.894	1.000	**0.921**
Adoption of infection-prevention measures	1.000	0.894	0.894	1.000	**0.947**
Use of pain-assessment scales	1.000	0.894	1.000	1.000	**0.974**
Adoption of comfort measures	0.894	0.894	1.000	1.000	**0.947**
Transfer performed	0.894	0.684	1.000	0.894	**0.868**
**CVR-S**	**0.951**	**0.865**	**0.918**	**0.966**	**0.925**

*CVR I - Content Validity Ratio of each item; CVR S - Total Content Validity Ratio; SSVV - vital signs; CT - computed tomography; Echo - echocardiogram; US - ultrasound; NB - newborn; PIV - peripheral intravenous access; PICC - peripherally inserted central catheter; MV - mechanical ventilation; NIV - non invasive ventilation.*

Regarding the CVR of the “Checking” section, all items presented agreement indices within the established parameters, except for the item “occurrence of an adverse event”, which obtained a CVR I of 0.684 for the attribute Clarity, a value below the recommended cutoff point (0.737). Nevertheless, the item was retained in the current version of the checklist, as the specialists’ suggestions indicated the need for specifying the type of adverse event (Table 2).

**Table 2:** Concordance Index (objectivity, clarity, simplicity, and relevance) and Content Validity Ratio of the “Checking” section of the checklist by attributes and overall, according to the evaluation of the specialists (N=19), Piauí, Brazil, 2021

Checking	Objectivity	Clarity	Simplicity	Relevance	CVR-I
Newborns identified	1.000	0.894	0.894	1.000	**0.947**
Beds/incubators identified	1.000	1.000	1.000	0.894	**0.974**
Newborns with child health card completed	0.789	0.789	0.894	0.789	**0.815**
Caregivers informed about unit routines	1.000	1.000	1.000	1.000	**1.000**
Caregivers informed about the indication for hospitalization	0.894	1.000	1.000	1.000	**0.974**
Medical records with updated prescriptions	1.000	1.000	1.000	1.000	**1.000**
Medical records with scheduled medications	0.894	0.894	0.894	0.894	**0.894**
Newborns with progress notes recorded	1.000	1.000	1.000	0.894	**0.974**
Newborns with allergies	0.894	0.789	0.789	0.894	**0.842**
Newborns with comorbidities	0.894	0.789	0.894	0.894	**0.868**
Newborn’s skin assessed	1.000	1.000	1.000	1.000	**1.000**
Functioning intravenous devices	1.000	0.894	1.000	1.000	**0.974**
Dated infusion sets	1.000	0.894	0.894	1.000	**0.947**
Newborn with oxygenation/ventilation/CPAP	1.000	1.000	1.000	1.000	**1.000**
Newborn with drains/catheters/tubes	1.000	0.894	0.894	0.894	**0.921**
Drains/catheters/tubes assessed	1.000	0.894	0.789	0.894	**0.894**
Incubator/crib temperature adequate	1.000	0.789	0.894	0.894	**0.894**
Medications checked	0.894	0.894	0.789	1.000	**0.894**
Occurrence of adverse event	0.789	0.684	1.000	1.000	**0.868**
Reports completed	0.894	0.894	0.789	0.894	**0.868**
Daily census completed	1.000	1.000	1.000	0.789	**0.947**
**CVR-S**	**0.950**	**0.904**	**0.924**	**0.934**	**0.928**

*CVR I - Content Validity Ratio of each item; CVR S - Total Content Validity Ratio; CPAP - continuous positive airway pressure; NB - newborn.*

Regarding the CVR of the “Notes” section, only the item “transfer required” presented agreement indices for Objectivity (0.684) and Clarity (0.574) below the expected threshold. However, it was not excluded, as the specialists’ suggestion to modify the item to “in hospital referral (exams/surgeries) required” and “inter hospital referral (exams/surgeries) required” was accepted, and the revised items were submitted to the second evaluation round, whose indices will be presented later. Furthermore, both the CVR I and CVR S were within the established agreement standards (Table 3).

**Table 3:** Concordance Index (objectivity, clarity, simplicity, and relevance) and Content Validity Ratio of the “Notes” section of the checklist by attributes and overall, according to the evaluation of the specialists (N=19), Piauí, Brazil, 2021

Notes	Objectivity	Clarity	Simplicity	Relevance	CVR-I
Physical examination recorded	1.000	1.000	0.894	1.000	**0.974**
Vital signs recorded	1.000	1.000	1.000	1.000	**1.000**
Intravenous hydration recorded	1.000	1.000	1.000	0.894	**0.974**
Fluid balance recorded	1.000	1.000	0.894	1.000	**0.974**
Test results recorded	0.894	1.000	1.000	1.000	**0.974**
Diet recorded	1.000	1.000	0.894	1.000	**0.974**
Newborn’s bath recorded	0.984	1.000	1.000	0.789	**0.943**
Dressings recorded	1.000	1.000	1.000	1.000	**1.000**
Drains/catheters/tubes recorded	1.000	0.894	1.000	0.894	**0.947**
Drain, catheter, and tube change/cleaning recorded	1.000	1.000	1.000	1.000	**1.000**
Peripheral IV insertion/change recorded	1.000	1.000	1.000	1.000	**1.000**
PICC insertion/change recorded	1.000	1.000	1.000	1.000	**1.000**
Insertion/change/cleaning of mechanical and non-invasive ventilation interfaces recorded	1.000	1.000	1.000	1.000	**1.000**
Adverse event reported	1.000	0.894	0.894	1.000	**0.947**
Intercurrences recorded	0.894	1.000	1.000	1.000	**0.974**
Transfer required	0.684	0.578	1.000	0.894	**0.789**
**CVR-S**	**0.966**	**0.960**	**0.974**	**0.967**	**0.967**

*CVR I - Content Validity Ratio of each item; CVR S - Total Content Validity Ratio; VS - vital signs; NB - newborn; PIV - peripheral intravenous access; PICC - peripherally inserted central catheter; MV - mechanical ventilation; NIV - non invasive ventilation.*

The analysis of the general aspects of the checklist revealed high levels of agreement among the specialists. The CVR values for each category were: CVR S = 0.925 for Procedures, 0.928 for Checking, and 0.967 for Notes, resulting in an overall CVR S of 0.940. These results indicate that the checklist items adequately meet the proposed objectives, confirming its potential to promote effective communication among NICU nursing professionals, the target audience for whom the checklist was developed.

Based on the specialists’ suggestions, gaps were identified in the initial content of the checklist, leading to the inclusion of 32 new items, proportionally redistributed across the three categories according to the logic of integrating actions, checking, and documentation. Specifically, 11 items were added to the Procedures category, five to the Checking category, and 16 to the Notes category, respecting the interdependence among categories - every procedure performed must necessarily be checked and documented.

Additionally, the attribute Breadth was assessed, corresponding to the checklist’s ability to comprehensively cover the phenomenon under study. According to 18 of the 19 specialists (94.73%), the checklist demonstrated adequate breadth to evaluate communication among nursing professionals in the NICU context. All items were considered objective, clear, simple, and relevant.

Due to the changes made and the inclusion of new items, a second evaluation round was conducted with the specialists in order to validate the additional content. The item “Inter hospital referral (exams/surgeries) performed” obtained 90% agreement, below the critical CVR value of 1.000. However, it was retained in the checklist because it presented CVR I and CVR S values equal to 1.000 within the Notes category, ensuring its relevance in the context of documenting care information.

All items in the Checking category reached 100% agreement for the attribute Relevance and were therefore considered valid. In the Notes category, although most items showed agreement above 80%, the item “Fall prevention measures recorded” did not reach the critical value for the attribute Relevance and was therefore excluded from the checklist.

After completing the two evaluation rounds and consolidating the statistical analyses (CVR I and CVR S), the checklist (final version) consisted of 84 items, distributed as follows: 31 items in the Procedures category, 23 in Checking, and 30 in Nursing Notes. The checklist achieved an average (global) CVR of 0.948 and is available in Supplement 1.

## DISCUSSION

The development and validation of checklists aimed at patient safety in high complexity settings, such as NICUs, are essential initiatives for improving the quality of care. In this study, the creation of a checklist to support effective communication among nursing professionals represents a significant advancement in systematizing safety strategies directed toward neonatal care. The results demonstrate that the checklist presents attributes of representativeness, relevance, and clarity within the content domains, which are widely used criteria in content validity assessment and considered fundamental to this process^(29)^.

The initial construction of the checklist was based on visual and structural guidelines designed to enhance readability, comprehension, and applicability. The choice of a simple, structured, and systematized layout, combined with the logical categorization of items into Procedures, Checking, and Notes, was intended to facilitate the organization of clinical data and reinforce the interdependence between care actions and documentation-an aspect widely recognized as essential for continuity of care^(30)^.

The first stage of this study involved an ILR^(21)^, consultations with reference instruments on patient safety^(22,23)^, and input from nurses, with the aim of encompassing the various evaluative aspects necessary for constructing a reliable and valid checklist. This stage supported a clear and rational conceptual definition of the construct to be validated, based on its intended use, prior to the conceptual formulation. The validity of the construct therefore depended on a solid theoretical definition, and not exclusively on psychometric testing^(31)^.

The development of the checklist through a participatory, consultative approach integrated into the workflow contributed to prioritizing the real context in which the research was conducted. This was essential during the planning phase and will remain crucial in the implementation and analysis phases to be carried out in the future. In this way, a pragmatic approach was adopted to accelerate the translation of evidence into practice^(32)^.

In this context, the ILR played a guiding role by identifying knowledge gaps that informed the content of the checklist^(33)^, and the active participation of NICU nurses from the regional hospital in constructing the second version of the checklist helped align the content with everyday clinical practice. The participatory approach - which involved suggestions at different stages and practical testing of the checklist - strengthened the process and ensured alignment with the team’s needs, as evidenced in similar studies^(16,34)^.

In the validation stage, the geographic and academic diversity of the specialists added value to the content assessment, promoting a more comprehensive analysis of the checklist from multiple perspectives. The distribution of specialists across four Brazilian regions and seven states, although with predominance from the Northeast, contributed to sample heterogeneity - an essential aspect for cross cultural validation and applicability in different healthcare contexts^(35,36)^. Thus, the premise was followed that specialists should possess sufficient content expertise, representativeness, and theoretical grounding to provide a comprehensive evaluation of the checklist^(37)^.

Although most items presented satisfactory CVR I and CVR S values - above the baseline according to the parameters in the literature^(28,29)^ - some items fell below the cutoff point for the attributes Clarity and Relevance. The decision to retain them for re evaluation, based on the specialists’ qualitative contributions and on values still within the acceptable range^(38)^, reflects the commitment to clinical pertinence and coherence with the checklist’s objectives. This strategy aligns with approaches that prioritize usability in real world contexts^(28)^.

The expansion of the checklist with 32 new items redistributed across the original categories demonstrates its ability to evolve based on practical and theoretical evidence. This process addressed the need to avoid content under representation, ensuring that all essential elements of the construct were included. To achieve this, it was crucial to apply theory and expert judgment rigorously, ensuring coherence between the included elements, the study objectives, and the operational definitions adopted^(29)^.

In the second validation round, although the item “Inter hospital referral (exams/surgeries) performed” did not reach the critical CVR value for Relevance, it was retained based on its clinical importance and other statistical parameters. Conversely, the item “Fall prevention measures recorded” was excluded for not meeting the minimum requirements, demonstrating the methodological rigor adopted and the commitment to the checklist’s technical excellence. The exclusion of irrelevant content is essential to avoid threats to representativeness and to preserve the checklist’s content validity^(29)^.

The final version of the checklist, composed of 84 items (31 Procedures, 23 Checking items, and 30 Notes), achieved an overall CVR of 0.948, indicating an almost perfect level of agreement among the specialists. The analysis of the attribute Breadth showed that 94.73% of the evaluators considered the content comprehensive and representative of the complexity of communication in NICUs, supporting the relevance of the selected items^(38)^. The validation of the checklist was an essential step, allowing for a thorough assessment and refinement of the instrument, culminating in its third and final version. Considered adequate by the specialists, this version was recommended for use in NICUs, establishing itself as an effective tool for strengthening the communication competencies of nursing teams^(37)^.

Despite this, it is necessary to consider the applicability of the developed checklist in real world practice. A multifaceted intervention is proposed to support adherence and impact clinical outcomes^(39)^. The initial implementation should include preparation involving unit leadership, ensuring the availability of necessary resources prior to implementation, and training on the use of the checklist, which may be complemented by additional technical training. Ongoing support strategies may also include regular on site mentoring, group text messaging for problem solving, refresher training, and regular safety or quality review meetings^(40)^.

Furthermore, it is essential to ensure that the use of the checklist is not perceived by the nursing team merely as the addition of new tasks to their daily routine that increase workload, but rather as a strategy to reduce the occurrence of adverse events. To achieve this, healthcare institutions must foster a culture of safety and provide training for professionals to use the checklist effectively^(41)^.

### Study limitations

As a limitation of the present study, it is noted that the checklist has not yet been tested in a real clinical environment, a stage that is currently under development by the research team. Nevertheless, the content validation represents a significant advancement for nursing and for the healthcare field as a whole, expanding the possibilities for incorporating strategies aimed at promoting patient safety in neonatal settings.

### Contributions to the field of Nursing

The checklist developed in this study proves to be a promising instrument for improving the transition of care between shifts in NICUs, promoting continuity of care and standardization of processes. Its application may enhance communication among professionals^(16)^, reduce failures, and strengthen safe practices, as evidenced by studies highlighting the effectiveness of checklists in improving clinical outcomes^(42)^, especially in pediatric and high complexity settings^(18,43)^.

## CONCLUSIONS

The construction of the checklist to support effective communication of nursing care in the neonatal intensive care unit was grounded in three dimensions - theoretical, operational, and empirical - and supported by scientific evidence related to patient safety. The development process involved nurses directly engaged in clinical practice, while content validation was carried out by a group of specialists who considered the checklist appropriate and recommended its use after the incorporation of suggested adjustments. Thus, the adoption of this checklist, specifically designed for the neonatal environment, emerges as a relevant strategy to ensure a safer and more efficient handoff among nursing professionals. It is expected that the checklist will foster the development of nursing practices that contribute to the continuous improvement of care quality.

## Data Availability

The research data are available within the article.

## References

[1] Araújo GT, Loss IO, Guimarães EL. (2024). Evaluation of pain and vital signs in newborns undergoing physiotherapeutic interventions in a neonatal intensive care unit. BrJP.

[2] Shahkolahi Z, Irajpour A, Jafari-Mianaei S, Heidarzadeh M. (2022). Developing patient safety standards for health-care quality promotion in neonatal intensive care units: a mixed-methods Protocol. J Educ Health Promot.

[3] Brado L, Tippmann S, Schreiner D, Scherer J, Plaschka D, Mildenberger E, Kidszun A. (2021). Patterns of safety incidents in a neonatal intensive care unit. Front Pediatr.

[4] Albanese FB, Ventura DS, Perroud MW, Souza RN, Morau MV, Visacri MB (2025). Adverse events in the neonatal intensive care unit identified by triggers. Front Pharmacol.

[5] Caeymaex L, Lebeaux C, Roze JC, Danan C, Reynaud A, Jung C (2020). Study on preventing adverse events in neonates (SEPREVEN): a stepped wedge randomised controlled trial to reduce adverse event rates in the NICU. Medicine (Baltimore).

[6] Galvão Diniz C, Regne GRS, Silva DCZ, Corrêa ADR, Mata LRFD, Manzo BF. (2024). Elaboration and validity of admission and discharge checklists in Neonatal Intensive Care Units. Rev Gaucha Enferm.

[7] Gibson K, Smith A, Sharp R, Ullman A, Morris S, Esterman A. (2024). Adverse events associated with umbilical vascular catheters in the neonatal intensive care unit: a retrospective cohort study. Aust Crit Care.

[8] Wieke-Noviyanti L, Ahsan A, Sudartya TS. (2021). Exploring the relationship between nurses’ communication satisfaction and patient safety culture. J Public Health Res.

[9] Yoo J, Chung SE, Oh J. (2021). Safety Climate and organizational communication satisfaction among korean perianesthesiac unit nurses. J Perianesth Nurs.

[10] Cui Y, Wang Y, Liu H, Xu S, Zhang X. (2025). Exploring the correlation between patient safety culture and adverse medical events using failure mode and effect analysis (FMEA). Risk Manag Healthc Policy.

[11] Jaulin F, Lopes T, Martin F. (2021). Standardised handover process with checklist improves quality and safety of care in the postanaesthesia care unit: the postanaesthesia team handover trial. Br J Anaesth.

[12] Dietl JE, Derksen C, Keller FM, Lippke S. (2023). Interdisciplinary and interprofessional communication intervention: How psychological safety fosters communication and increases patient safety. Front Psychol.

[13] Li B, Yue L, Peng H, Chen X, Sohaib M, Peng B, Zhang T, Zou W. (2025). Analysis of the incidence and factors influencing medication administration errors among nurses: a retrospective study. J Clin Nurs.

[14] Suraya CS, bin Sansuwito T, Regidor D, Wisuda AC (2024). Effective communication in nursing: a comprehensive systematic review of best practices. JNSR.

[15] Gunnels MS, Thompson SL, Jenifer Y. (2024). Use of rounding checklists to improve communication and collaboration in the adult intensive care unit: an integrative review. Crit Care Nurse.

[16] Manzo BF, Silva DCZ, Fonseca MP, Tavares IVR, Marcatto JO, Mata LRF (2023). Content validity of a Safe Nursing Care Checklist for a neonatal unit. Nurs Crit Care.

[17] Concha-Torre A, Díaz Alonso Y, Álvarez Blanco S, Vivanco Allende A, Mayordomo-Colunga J, Fernández Barrio B. (2020). [The checklists: A help or a hassle?]. An Pediatr.

[18] Saraiva COPO, Andrade FB, Chiavone FBT, Barbosa ML, Medeiros SG, Souza NL (2022). Neonatal patient safety assessment: construction and validation of a protocol and a checklist. Acta Paul Enferm.

[19] Brito MAB. (2021). Elaboração e validação de um checklist sobre comunicação segura dos cuidados de enfermagem na unidade neonatal.

[20] Pasquali L. (1997). Princípios de elaboração de escalas psicológicas.

[21] Brito MA, Carneiro CT, Bezerra MAR, Rocha RC, Rocha SS. (2022). Effective communication strategies among health professionals in neonatology: an integrative review. Enf Global.

[22] Ministério da Saúde (BR) (2014). Documento de referência para o Programa Nacional de Segurança do Paciente.

[23] Joint Commission International (JCI) (2017). International patient safety goals.

[24] Taylor MJ, McNicholas C, Nicolay C, Darzi A, Bell D, Reed JE. (2014). Systematic review of the application of the plan-do-study-act method to improve quality in healthcare. BMJ Qual Saf.

[25] Guimarães HCQCP, Pena SB, Lopes JL, Lopes CT, Barros ALBL. (2016). Experts for validation studies in nursing: new proposal and selection criteria. Int J Nurs Knowl.

[26] LoBiondo-Wood G, Haber J. (2014). Nursing Research: methods and critical appraisal for evidence-based practice. J Nurs Regul.

[27] Ayre C, Scally AJ. (2014). Critical values for lawshe’s content validity ratio: revisiting the original methods of calculation. MECD.

[28] Polit DF, Beck CT, Owen SV. (2007). Is the CVI an acceptable indicator of content validity? appraisal and recommendations. Res Nurs Health.

[29] Almanasreh E, Moles R, Chen TF. (2019). Evaluation of methods used for estimating content validity. Res Social Adm Pharm.

[30] Escamilla-Ocañas CE, Torrealba-Acosta G, Mandava P, Qasim MS, Gutiérrez-Flores B, Bershad E (2022). Implementation of systematic safety checklists in a neurocritical care unit: a quality improvement study. BMJ Open Qual.

[31] Smith MR, Munnik E. (2023). The development of the conceptual construct validity appraisal checklist. Afr J Psychol Assess.

[32] Flemington T, Hort J, Marks S, Tankel A, Tzioumi D, Fraser J. (2025). Implementation and evaluation of a hospital-based electronic checklist for the clinical assessment of child maltreatment. Glob Implement Res Appl.

[33] Maciel MPR, Costa LMA, Sousa KHJF, Oliveira ADS, Amorim FCM, Moura LKB (2022). Construction and validation of a serious game about human. papillomavirus infection.

[34] Ventura-Silva JMA, Silva Martins MMFPD, Trindade LL, Faria ADCA, Barros SCDC, Melo RMDC (2023). Nurses’ work methods assessment scale: a study of content validation. Rev Bras Enferm.

[35] Leonhardt GB, Silva GBD, Prates GK, Canabarro ST, Souza LM. (2025). Construction and validation of a video on the insertion of gastric and enteral tubes in children. Rev Latino-Am Enfermagem.

[36] Day SW, Sullivan CE, Morrissey L, Abramovitz L, Segovia L, Punjwani R (2021). Development and content validation of an instrument to measure baseline standards for pediatric oncology nursing in lowand middle-income countries. J Pediatr Oncol Nurs.

[37] Atashzadeh-Shoorideh F, Shirinabadi Farahani A, Pishgooie AH, Babaie M, Hadi N, Beheshti M (2022). A comparative study of patient safety in the intensive care units. Nurs Open.

[38] Ahmad KA, Idris I. A (2025). CEFR-Informed English Test: how content validity index and content validity ratio are used for content validation. EJOSS.

[39] Sousa KM, Saturno-Hernández PJ, Rosendo TMSS, Freitas MR, Molina RL, Medeiros WR Impact of the implementation of the WHO Safe Childbirth Checklist on essential birth practices and adverse events in two Brazilian hospitals: a before and after study. BMJ Open.

[40] Molina RL, Benski AC, Bobanski L, Tuller DE, Semrau KEA. (2021). Adaptation and implementation of the WHO Safe Childbirth Checklist around the world. Implement Sci Commun.

[41] Custódio RJM, Kapassi LB, Alves DT, Barros AF, Melo MC, Boeckman LMM (2021). Perception of nursing professionals on the use of the Safe Delivery Checklist. Cogitare Enferm.

[42] White MC, Peven K, Clancy O, Okonkwo I, Bakolis I, Russ S (2021). Implementation strategies and the uptake of the World Health Organization surgical safety checklist in lowand middle-income countries: a systematic review and meta-analysis. Ann Surg.

[43] Melo AVOG, Noronha RDB, Nascimento MAL. (2022). Use of checklist for safe care of hospitalized children. Rev Enferm UERJ.

