# Supplementary material for: Construction and content validation of a checklist for effective communication of nursing care to newborns
Source: Rev Bras Enferm. 2026 Sep 21;79:e20250407. doi: 10.1590/0034-7167-2025-0407 (PMC13592785; doi:10.1590/0034-7167-2025-0407)
Supplement: Translation

# Translation: Construção e validação do conteúdo de *checklist* para comunicação efetiva dos cuidados de enfermagem aos recém-nascidos

**DOI:** 10.1590/0034-7167-2025-0407pt

**Authors:** Mychelangela de Assis Brito, Cristianne Teixeira Carneiro, Ruth Cardoso Rocha, José Wicto Pereira Borges, Maria Augusta Rocha Bezerra, Silvana Santiago da Rocha

**Affiliations:** I Universidade Federal do Piauí. Floriano, Piauí, Brasil; II Universidade Federal do Piauí. Teresina, Piauí, Brasil

**Keywords:** Recém-Nascido, Segurança do Paciente, Comunicação, Lista de Checagem, Estudo de Validação.

## Abstract

**Objetivos:**

construir e validar o conteúdo de um *checklist* para apoiar a comunicação dos cuidados de enfermagem aos recém-nascidos internados em unidade de terapia intensiva neonatal.

**Métodos:**

estudo metodológico desenvolvido em duas etapas. A primeira envolveu três dimensões: teórica, fundamentada por uma revisão integrativa da literatura; operacional, com a elaboração da versão inicial do *checklist*; e empírica, conduzida através do ciclo PDCA (*Plan, Do, Check, Act*), com a participação de 15 enfermeiras de uma unidade de terapia intensiva neonatal. A segunda etapa consistiu na validação de conteúdo, por 19 especialistas, utilizando-se a Razão de Validade de Conteúdo.

**Resultados:**

o *checklist* final foi composto por 84 itens, distribuídos em: 31 itens na categoria Procedimentos, 23 em Checagem e 30 em Anotações. A Razão de Validade de Conteúdo atingiu média global de 0,948.

**Conclusões:**

o *checklist* foi validado quanto ao seu conteúdo por consenso pelo comitê de especialistas, podendo ser utilizado na prática assistencial.

## INTRODUÇÃO

A unidade de terapia intensiva neonatal (UTIN) é um ambiente terapêutico de alta complexidade, destinado ao cuidado de recém-nascidos (RN) em situação de risco^(1)^, mas que apresenta significativa suscetibilidade à ocorrência de eventos adversos (EA) em razão da coexistência de múltiplas ameaças de natureza estrutural e processual^(2)^. Destacam-se entre esses EA erros de medicação, falhas em equipamentos, equívocos diagnósticos, problemas na comunicação entre profissionais, falhas de documentação e deficiências nas práticas de higiene^(3)^.

Aproximadamente metade dos RN internados em UTIN vivenciam pelo menos um EA^(4)^, os quais, em sua maioria, podem ser classificados como evitáveis ou provavelmente evitáveis^(3)^. Tais eventos estão entre as principais causas de lesões e mortalidade em pacientes neonatais^(5)^, o que torna evidente a necessidade de desenvolvimento e implementação de estratégias preventivas que visem à sua redução, promovendo, assim, a segurança do paciente^(6)^.

A adoção de mudanças na prática clínica, bem como a identificação e controle de fatores de riscos individuais e sistêmicos, são fundamentais para minimizar os EA em UTIN^(7)^. Nesse contexto, a comunicação destaca-se como pilar fundamental, sendo reconhecida como um dos principais determinantes na promoção da segurança do paciente^(8)^.

A comunicação efetiva é fundamental em ambientes hospitalares, onde diferentes profissionais de saúde atuam de forma integrada. Enfermeiros, em especial, desempenham papel central nesse processo, uma vez que interagem constantemente com diversas categorias profissionais^(9)^. A satisfação com a comunicação também interfere diretamente na qualidade da assistência prestada^(8)^, com muitos estudos demonstrando que falhas na comunicação interpessoal e entre equipes são responsáveis por grande parte dos EA evitáveis em serviços de saúde^(10,11)^.

Nota-se relação direta entre comunicação efetiva, trabalho em equipe e desempenho em segurança assistencial^(12)^, com procedimentos estruturados de comunicação sendo capazes de reduzir EA evitáveis em até 30% dos casos^(13)^. A comunicação efetiva compreende estratégias para assegurar a transmissão clara e precisa de informações, contribuindo para a redução de riscos e para o aumento da segurança tanto dos pacientes quanto dos profissionais envolvidos na assistência^(14)^. Em contrapartida, lacunas informacionais durante a passagem de plantão, ausência de protocolos padronizados, atividades multitarefas, distrações e pressão de tempo são apontados como fatores que comprometem a qualidade da comunicação^(11)^.

Diante disso, estudos recentes indicam que o uso de roteiros padronizados, como *checklists*, especialmente durante rondas interprofissionais, impactam positivamente os resultados clínicos^(11,15)^. No âmbito específico da enfermagem, o uso desses roteiros melhora a comunicação, reduz a ocorrência de eventos adversos e promove cuidados seguros e de qualidade em diferentes ambientes de cuidado^(16)^. Além de padronizar o cuidado e reduzir a variabilidade da transmissão das informações, os *checklists* promovem a eficácia do trabalho em equipe e a comunicação entre os profissionais de saúde^(17)^.

Embora existam avanços no uso de *checklists* em ambientes críticos, os estudos com foco específico na aplicabilidade desses instrumentos em UTIN ainda são escassos^(16,18)^, e há indicação da necessidade de pesquisas contínuas voltadas à adaptação dos *checklists* às particularidades das unidades neonatais e às demandas específicas dos recém-nascidos^(17)^. No âmbito internacional, diversos *checklists* de enfermagem foram desenvolvidos com o objetivo de promover a segurança dos cuidados prestados a RN hospitalizados em UTIN^(2,16)^, assim como no Brasil^(6,18)^. Contudo, até o presente momento, não foram identificados *checklists* sobre o tema validados para enfermeiros que atuam no cuidado a RN em UTIN que padronize, de forma específica, os diferentes aspectos da comunicação durante a passagem de plantão.

## Relevância do estudo

Este artigo é produto da Tese^(19)^ depositada no repositório institucional http://repositorio.ufpi.br:8080/xmlui/handle/123456789/324.

## OBJETIVOS

Construir e validar o conteúdo de um *checklist* para apoiar a comunicação dos cuidados de enfermagem aos recém-nascidos internados em UTIN.

## MÉTODOS

### Delineamento do estudo

Trata-se de um estudo metodológico conduzido em duas etapas: construção do *checklist* e validação de conteúdo. Ambas se fundamentam nos pressupostos da psicometria, que orientam o desenvolvimento de instrumentos de mensuração em saúde, especialmente na operacionalização do construto por meio de itens^(20)^. A pesquisa segue as diretrizes do *Standards for Quality Improvement Reporting Excellence* (SQUIRE 2.0) por ser uma abordagem para a melhoria da assistência à saúde.

### Procedimentos do estudo

As etapas do estudo foram estruturadas com procedimentos específicos de execução e coleta de dados. A Etapa 1, correspondente à construção do *checklist*, foi subdividida em três fases interdependentes: (1) dimensão teórica, (2) dimensão operacional e (3) dimensão empírica.

Na dimensão teórica, realizou-se uma revisão integrativa da literatura (RIL)^(21)^ sobre comunicação efetiva em saúde, com o objetivo de identificar indicadores empíricos relevantes para a elaboração dos itens do *checklist*. A pergunta de pesquisa foi estruturada com base na estratégia PICo - P (segurança do paciente), I (comunicação efetiva), Co (neonatologia) - formulada da seguinte forma: “Quais estratégias são utilizadas para uma comunicação efetiva entre os profissionais de saúde visando à segurança do recém-nascido hospitalizado na UTIN?”. A busca foi realizada via Portal da CAPES, com acesso pela Universidade Federal do Piauí (UFPI), abrangendo seis bases de dados.

Na dimensão operacional, elaborou-se a primeira versão do *checklist* com base nos achados da RIL e nas especificidades da prática assistencial em unidades neonatais. O desenvolvimento do *checklist* seguiu as diretrizes das Metas Internacionais de Segurança do Paciente da Organização Mundial da Saúde e do Programa Nacional de Segurança do Paciente^(22,23)^. Foram definidos o uso pretendido e a área de interesse do *checklist*. Em seguida, selecionaram-se os descritores e organizaram-se os itens por categorias, com definições conceituais claras. A redação dos itens foi objetiva e direta, favorecendo respostas rápidas pela equipe de enfermagem.

A dimensão empírica teve como finalidade avaliar a aplicabilidade da versão inicial do *checklist* à realidade assistencial. Para isso, adotou-se a metodologia do ciclo PDCA (*Plan, Do, Check, Act*), reconhecida por sua eficácia na promoção da melhoria contínua da qualidade e segurança em saúde^(24)^. Enfermeiros da UTIN do hospital regional participaram da elaboração da segunda versão do *checklist*, após apresentação da primeira versão pela equipe de pesquisa. Foram realizados grupos focais com os participantes, com o objetivo de integrar conhecimento científico e experiência prática. Essa fase possibilitou a inclusão, exclusão e modificação de itens, culminando na consolidação da segunda versão do *checklist*. Nessa ocasião, também foi aplicado um questionário de caracterização dos participantes.

Na Etapa 2, referente à validação do conteúdo do *checklists*, a coleta de dados foi realizada virtualmente, por meio de formulário no *Google Forms* ^®^ enviado via *e-mail* e *WhatsApp Messenger* ^®^. O link incluía a carta convite, a solicitação de aceite (com Termo de Consentimento Livre e Esclarecido - TCLE) e os formulários para caracterização dos participantes e avaliação dos itens do *checklist*. Um prazo de 15 dias foi estipulado para devolução dos materiais, respeitando o cronograma da pesquisa.

### Local da coleta de dados

As fases 1 (dimensão teórica) e 2 (operacional) da construção do *checklist* (Etapa 1) foram realizadas nos laboratórios de ensino da Universidade Federal do Piauí (UFPI), em um campus do interior, com participação de docentes do curso de Bacharelado em Enfermagem. A terceira fase dessa etapa, dimensão empírica, ocorreu em uma sala privativa de um hospital regional no estado do Piauí. A validação do conteúdo do *checklist* (Etapa 2) foi conduzida em ambiente virtual, utilizando a Plataforma Lattes^®^, *Google Drive* ^®^ e *Google Forms* ^®^.

### Período

O estudo foi desenvolvido entre os meses de março de 2020 e agosto de 2021.

### População

Para a fase operacional da Etapa 1, a população foi composta por todos os 27 enfermeiros atuantes na área de neonatologia do hospital regional, sendo seis deles lotados na Unidade Neonatal, 15 no Alojamento Conjunto e seis na UTIN. Na Etapa 2, os especialistas foram recrutados em todo o território brasileiro, via Plataforma Lattes^®^, que reúne, de forma aberta, o currículo de pesquisadores (Currículo Lattes) e informações de grupos de pesquisa e instituições no país.

### Critérios de seleção

Na Etapa 1, para construção e operacionalização do *checklist*, os especialistas foram selecionados a partir dos seguintes critérios de elegibilidade: atuar na Unidade Neonatal, Alojamento Conjunto e/ou Unidade de Terapia Intensiva Neonatal (UTIN), e com experiência na UTIN. Foram excluídas 12 participantes pelos seguintes motivos: uma enfermeira estava de férias, duas de licença médica, duas não retornaram o convite para participação das reuniões, duas não participaram de todas as reuniões, duas não devolveram os instrumentos após a realização do ciclo e três se recusaram a participar por alegarem experiência apenas na obstetrícia.

Na Etapa 2, validação do constructo, os especialistas foram recrutados de duas formas. Incialmente realizou-se busca na Plataforma Lattes^®^, utilizando-se os termos “neonatologia”, “tecnologias”, “tecnologias em saúde”, “tecnologias em enfermagem”, “segurança do paciente” e “estudos de validação” no campo “Assunto”. Os filtros aplicados incluíram formação acadêmica (todas), área de atuação (ciências da saúde) e idioma (português). Os currículos dos candidatos foram analisados quanto à experiência prática e acadêmica, participação em projetos de pesquisa, produção científica relevante e atuação nas temáticas de interesse. A seleção dos especialistas seguiu critérios que priorizam a experiência clínica em estudos de validação na área da enfermagem^(25)^.

Aos especialistas que responderam o instrumento de coleta de dados foi solicitada a indicação de dois pesquisadores para participação da validação do material por meio da técnica de amostragem em rede (ou “bola de neve”), método apropriado para a identificação de profissionais com perfis altamente específicos, de difícil localização por métodos convencionais^(26)^.

Os critérios de inclusão para os especialistas foram: ser profissional da saúde, atuar no Brasil e alcançar, no mínimo, seis pontos nos Critérios para seleção dos *expertises* na área^(25)^, considerando experiência de pelo menos quatro anos na área específica (quatro pontos), experiência de pelo menos um ano no ensino da área específica (três pontos), experiência em pesquisa com artigos publicados em periódicos de referência na área específica (dois pontos), participação de pelo menos dois anos em um grupo de pesquisa na área específica (dois pontos), doutorado na área específica (dois pontos), mestrado na área específica (um ponto), e residência e/ou especialização na área específica (um ponto). As áreas específicas foram definidas como “tecnologias em saúde e/ou enfermagem, neonatologia, segurança do paciente e estudos de validação”. Como critério de exclusão, adotou-se não possuir como língua materna o português do Brasil, por ser esse o idioma do *checklist*.

### Definição da amostra

Na Etapa 1, a amostra por conveniência foi constituída por 15 enfermeiros. Na Etapa 2, a amostra de especialistas foi estipulada considerando o mínimo de seis e o máximo de 20 especialistas^(20)^, tendo contribuído 19 participantes.

### Variáveis do estudo

Para caracterização dos enfermeiros, foram coletadas as variáveis sexo, idade, escolaridade, titulação, tempo de formação e de atuação em neonatologia, setor de atuação, e capacitação em neonatologia e segurança do paciente. Para os especialistas, consideraram-se sexo, idade, procedência geográfica, formação, titulação e experiência nas áreas do estudo. Quanto à validação do *checklist*, foram avaliados os atributos psicométricos de objetividade, clareza, simplicidade e relevância.

### Instrumentos utilizados para a coleta de dados

Na Etapa 1, foram aplicados dois instrumentos: um questionário sociodemográfico e um roteiro de análise da primeira versão do *checklist*, com campos para sugestões. Na Etapa 2, utilizaram-se dois instrumentos: um questionário sociodemográfico e um formulário de avaliação dos itens do *checklist*, baseados em quatro dos 12 atributos psicométricos: Objetividade, Clareza, Simplicidade e Relevância^(20)^. As respostas foram classificadas em: 1 = sim, 2 = não, 0 = não sei opinar.

### Tratamento e análise dos dados

Os dados foram organizados no *Excel for Windows* (versão 2110). Foram utilizadas frequências para variáveis categóricas e medidas descritivas (média, mínimo, máximo, desvio padrão) para variáveis contínuas.

A validação dos itens utilizou a Razão de Validade de Conteúdo (CVR), conforme fórmula: CVR = (ne - N/2) / (N/2), em que “ne” é o número de especialistas que consideraram o item essencial e “N” é o total de especialistas^(27)^. Consideraram-se válidos os itens com CVR-I ≥ 0,737. Itens com pontuação inferior em “Relevância” foram excluídos - os demais foram revisados com base nas sugestões dos especialistas e submetidos a uma segunda rodada de validação. Adicionalmente, foi adotado o Índice de Concordância, com ponto de corte de 0,80^(20)^.

Na segunda rodada, utilizou-se um grupo reduzido (5 especialistas) para reavaliar itens ajustados^(28)^. Foram selecionados especialistas que forneceram contribuições qualitativas relevantes. O ponto de corte adotado foi CVR = 1,000^(27)^.

### Aspectos éticos

O estudo foi conduzido de acordo com as diretrizes de ética nacionais e internacionais e aprovado pelo Comitê de Ética em Pesquisa da Universidade Federal do Piauí. O Consentimento Livre e Esclarecido foi obtido dos especialistas nas duas coletas de dados de forma *online*. O parecer está anexado à presente submissão. Os documentos físicos foram guardados em um armário trancado no gabinete da pesquisadora responsável, enquanto os ficheiros digitais foram armazenados em pastas protegidas por palavra-passe no seu computador, com acesso restrito exclusivamente à equipe de pesquisa. Em todas as etapas do estudo, foi assegurado o anonimato dos participantes, tanto nas atividades presenciais como nas realizadas virtualmente.

## RESULTADOS

### Primeira etapa: construção do *checklist*

Participaram dessa etapa 15 enfermeiras. O perfil das participantes revelou que todas eram do sexo feminino (100%), com idades entre 27 e 41 anos (média de 33,5 ± 4,5 anos). O tempo de formação variou entre dois e 12 anos, com média de 7,7 ± 3,0 anos. No que se refere à titulação, 13 (86,7%) possuíam pós-graduação *Lato Sensu* e apenas duas (13,3%) eram somente graduadas. O tempo médio de atuação na área neonatal foi de 2,8 ± 2,1 anos. Em relação aos setores de atuação, sete enfermeiras (46,7%) atuavam no alojamento conjunto, três (20%) na neonatologia e cinco (33,3%) na UTIN. Apenas quatro enfermeiras (26,7%) relataram possuir especialização em Neonatologia e quatro (26,7%) indicaram formação específica em segurança do paciente.

A construção da primeira versão do *checklist* sobre comunicação efetiva entre profissionais de enfermagem voltada à segurança do paciente na UTIN seguiu diretrizes de organização visual e de conteúdo claras. O *checklist* foi formatado em fonte Times New Roman, cor preta, tamanho 12, distribuído em duas páginas com *layout* em tons de cinza. Estruturalmente, o *checklist* foi dividido em duas seções: a primeira, intitulada *“Finalidades do checklist e orientações de preenchimento”*, e a segunda, composta pelos itens a serem avaliados. Esta última seção foi disposta em duas colunas: *“Itens a serem checados”* e *“Respostas”*, com as opções de resposta: Sim, Não e Não se aplica (N/A). Os itens foram agrupados em três categorias principais: Anotações (9 itens), Checagem (17 itens) e Procedimentos (15 itens), totalizando 41 itens.

A versão revisada do *checklist* resultou em um total de 60 itens, redistribuídos nas categorias Procedimentos (22 itens), Checagem (21 itens) e Anotações (16 itens). Todos os itens sugeridos pelas participantes foram incorporados, considerando sua relevância para a prática assistencial e o contexto específico da UTIN. A segunda versão do *checklist*, agora mais abrangente e aderente à realidade clínica, foi submetida à etapa seguinte de validação de conteúdo.

### Segunda etapa: validação do *checklist*

A análise de conteúdo do *checklist* foi realizada em duas rodadas sucessivas, conforme preconizado em estudos metodológicos voltados à validação de instrumentos na área da saúde. Na primeira rodada, participaram 19 especialistas brasileiros, com idades entre 25 e 68 anos (média de 37,7 ± 9,2 anos), sendo a maioria do sexo feminino (n = 15; 78,9%), e oriundos de quatro das cinco regiões brasileiras (Norte, Nordeste, Sul e Sudeste), predominantemente da região Nordeste (n = 16; 84,2%), e de sete estados (RR, PI, MA, CE, PE, PR, SP), com destaque para o estado do Piauí (n = 11; 57,9%). A maioria dos participantes era graduado em Enfermagem (n = 16; 84,2%) e apresentava tempo de formação entre três e 45 anos (média de 13,5 ± 8,9 anos). Vale destacar que participaram graduados em Medicina (n=2; 10,05%) e em Fisioterapia (n=1; 5,3%). Quanto à titulação, 10 especialistas (52,6%) possuíam mestrado em áreas relacionadas à temática do estudo, como neonatologia, segurança do paciente, tecnologias em saúde e enfermagem, e estudos de validação.

O instrumento de avaliação enviado aos especialistas foi estruturado de forma a permitir comentários e sugestões em diferentes dimensões do *checklist:* título, finalidades e orientações de preenchimento, organização por categorias (Procedimentos, Checagem e Anotações) e *layout*. Em relação ao título, 4 especialistas (21,05%) fizeram observações. Quanto às orientações de preenchimento, foram registradas contribuições de 3 avaliadores (15,78%). A estrutura categorial do *checklist* recebeu sugestões de 4 especialistas (21,05%), enquanto aspectos relativos ao *layout* foram comentados por apenas 2 participantes (10,52%).

No que se refere à CVR dos itens da categoria “Procedimentos”, os resultados obtidos - tanto individuais (CVR-I) quanto da escala (CVR-S) - estiveram dentro dos parâmetros de concordância estabelecidos na literatura, conforme demonstrado na Tabela 1. No entanto, três itens apresentaram índices de concordância individual inferiores ao valor crítico (0,737): “drenos/cateteres/sondas realizados” e “transferência realizada” em relação ao atributo Clareza, e “banho do RN realizado” quanto à Relevância, com CVR-I de 0,684 cada. Esses itens foram alterados, conforme sugestões fornecidas pelos especialistas, e encaminhados para reavaliação na segunda rodada.

**Tabela 1:** Índice de Concordância (objetividade, clareza, simplicidade e relevância) e Razão de Validade de Conteúdo da seção “Procedimentos” do *checklist* por atributos e geral conforme avaliação dos especialistas (N=19), Piauí, Brasil, 2021

Procedimentos	Objetividade	Clareza	Simplicidade	Relevância	CVR-I
Exame físico realizado	1,000	1,000	0,894	1,000	**0,974**
SSVV verificados	0,894	0,894	0,789	0,894	**0,868**
Hidratação venosa realizada	0,894	0,789	0,894	1,000	**0,894**
Balanço hídrico realizado	1,000	1,000	1,000	1,000	**1,000**
Medicações realizadas	0,894	0,789	0,789	1,000	**0,868**
Dieta realizada	1,000	0,894	0,789	1,000	**0,921**
Exames Laboratoriais realizados	1,000	0,894	1,000	1,000	**0,974**
Exames de Imagem (Raio X, TC, ECO, USG) realizados	1,000	0,894	1,000	1,000	**0,974**
Procedimentos cirúrgicos realizados	0,894	1,000	1,000	1,000	**0,974**
Banho do RN realizado	0,894	0,894	0,789	0,684	**0,815**
Curativos realizados	0,894	0,894	1,000	0,894	**0,921**
Drenos/cateteres/sondas realizados	0,789	0,684	1,000	0,894	**0,842**
Troca/limpeza de dreno, cateter e sonda realizados	1,000	0,894	0,894	1,000	**0,947**
Inserção/Troca de AVP realizada	1,000	0,894	0,789	1,000	**0,921**
Inserção/Troca de PICC realizada	1,000	0,894	0,894	1,000	**0,947**
Inserção/Troca/limpeza da garganta da VM e VNI realizada	1,000	0,789	0,894	1,000	**0,921**
Adoção de medidas para prevenção de quedas	1,000	0,789	1,000	1,000	**0,947**
Adoção de medidas para prevenção de lesão por pressão	1,000	0,789	0,894	1,000	**0,921**
Adoção de medidas para prevenção de infecção	1,000	0,894	0,894	1,000	**0,947**
Adoção de escalas para avaliação da dor	1,000	0,894	1,000	1,000	**0,974**
Adoção de medidas de conforto	0,894	0,894	1,000	1,000	**0,947**
Transferência realizada	0,894	0,684	1,000	0,894	**0,868**
**CVR-S**	**0,951**	**0,865**	**0,918**	**0,966**	**0,925**

*CVR-I - Razão de Validade de Conteúdo de cada Item; CVR-S - Razão de Validade de Conteúdo Total; SSVV - sinais vitais; TC - tomografia computadorizada; ECO - ecocardiograma; USG - ultrassonografia; RN - recém-nascido; AVP - acesso venoso periférico; PICC - cateter central de inserção periférica; VN - ventilação mecânica; VNI - ventilação não invasiva.*

No que diz respeito à CVR da seção “Checagem”, observou-se que todos os itens apresentaram índices de concordância dentro dos parâmetros estabelecidos, com exceção do item “ocorrência de evento adverso”, que obteve um CVR-I de 0,684 para o atributo Clareza, valor inferior ao ponto de corte recomendado (0,737). Apesar disso, o item foi mantido na versão atual do *checklist*, visto que a sugestão dos especialistas indicava a necessidade de especificação quanto ao tipo de evento adverso (Tabela 2).

**Tabela 2:** Índice de Concordância (objetividade, clareza, simplicidade e relevância) e Razão de Validade de Conteúdo da seção “Checagem” do *checklist* por atributos e geral conforme avaliação dos especialistas (N=19), Piauí, Brasil, 2021

Checagem	Objetividade	Clareza	Simplicidade	Relevância	CVR-I
RN identificados	1,000	0,894	0,894	1,000	**0,947**
Leitos/berços identificados	1,000	1,000	1,000	0,894	**0,974**
RN com cartão da criança preenchido	0,789	0,789	0,894	0,789	**0,815**
Acompanhantes informados sobre rotina do setor	1,000	1,000	1,000	1,000	**1,000**
Acompanhantes informados sobre a indicação de internação	0,894	1,000	1,000	1,000	**0,974**
Prontuários com prescrição atualizada	1,000	1,000	1,000	1,000	**1,000**
Prontuários com medicações aprazadas	0,894	0,894	0,894	0,894	**0,894**
RN evoluídos	1,000	1,000	1,000	0,894	**0,974**
RN com alergia	0,894	0,789	0,789	0,894	**0,842**
RN com comorbidades	0,894	0,789	0,894	0,894	**0,868**
Pele do RN avaliada	1,000	1,000	1,000	1,000	**1,000**
Dispositivos intravenosos funcionantes	1,000	0,894	1,000	1,000	**0,974**
Equipos datados	1,000	0,894	0,894	1,000	**0,947**
RN com oxigenação/ventilação/CPAP	1,000	1,000	1,000	1,000	**1,000**
RN com drenos/cateteres/sondas	1,000	0,894	0,894	0,894	**0,921**
Drenos/cateteres/sondas avaliados	1,000	0,894	0,789	0,894	**0,894**
Temperatura incubadora/berço adequada	1,000	0,789	0,894	0,894	**0,894**
Medicações checadas	0,894	0,894	0,789	1,000	**0,894**
Ocorrência de Evento Adverso	0,789	0,684	1,000	1,000	**0,868**
Relatórios preenchidos	0,894	0,894	0,789	0,894	**0,868**
Censo diário preenchido	1,000	1,000	1,000	0,789	**0,947**
**CVR-S**	**0,950**	**0,904**	**0,924**	**0,934**	**0,928**

*CVR-I - Razão de Validade de Conteúdo de cada Item; CVR-S - Razão de Validade de Conteúdo Total; CPAP - pressão positiva contínua de vias aéreas; RN - recém-nascido.*

Quanto à CVR da seção “Anotações”, apenas o item “transferência requerida” apresentou um índice de concordância para Objetividade (0,684) e Clareza (0,574) abaixo da média esperada. Porém, ele não foi excluído, uma vez que foi acatada a sugestão de modificação do item para “encaminhamento intra-hospitalar (exames/cirurgias) requerido” e “encaminhamento inter-hospitalar (exames/cirurgias) requerido”, sendo submetido à segunda rodada de avaliação, cujos índices serão apresentados posteriormente. Ademais, o CVR-I e CVR-S estão dentro do padrão de concordância estabelecidos (Tabela 3).

**Tabela 3:** Índice de Concordância (objetividade, clareza, simplicidade e relevância) e Razão de Validade de Conteúdo da seção “Anotações” do *checklist* por atributos e geral conforme avaliação dos especialistas (N=19), Piauí, Brasil, 2021

Anotações	Objetividade	Clareza	Simplicidade	Relevância	CVR-I
Exame físico registrado	1,000	1,000	0,894	1,000	**0,974**
SSVV registrados	1,000	1,000	1,000	1,000	**1,000**
Hidratação venosa registrada	1,000	1,000	1,000	0,894	**0,974**
Balanço hídrico registrado	1,000	1,000	0,894	1,000	**0,974**
Resultados de exames registrados	0,894	1,000	1,000	1,000	**0,974**
Dieta registrada	1,000	1,000	0,894	1,000	**0,974**
Banho do RN registrado	0,984	1,000	1,000	0,789	**0,943**
Curativos registrados	1,000	1,000	1,000	1,000	**1,000**
Drenos/cateteres/sondas registrados	1,000	0,894	1,000	0,894	**0,947**
Troca/limpeza de dreno, cateter e sonda registrados	1,000	1,000	1,000	1,000	**1,000**
Inserção/Troca de AVP registrada	1,000	1,000	1,000	1,000	**1,000**
Inserção/Troca de PICC realizada	1,000	1,000	1,000	1,000	**1,000**
Inserção/Troca/limpeza da garganta da VM e VNI registrada	1,000	1,000	1,000	1,000	**1,000**
Evento adverso notificado	1,000	0,894	0,894	1,000	**0,947**
Intercorrências registradas	0,894	1,000	1,000	1,000	**0,974**
Transferência requerida	0,684	0,578	1,000	0,894	**0,789**
**CVR-S**	**0,966**	**0,960**	**0,974**	**0,967**	**0,967**

*CVR-I - Razão de Validade de Conteúdo de cada Item; CVR-S - Razão de Validade de Conteúdo Total; SSVV - sinais vitais; RN - recém-nascido; AVP - acesso venoso periférico; PICC - cateter central de inserção periférica; VN - ventilação mecânica; VNI - ventilação não invasiva.*

A análise dos aspectos gerais do *checklist* revelou elevados índices de concordância entre os especialistas. Os valores da CVR para cada categoria foram: CVR-S = 0,925 para Procedimentos, 0,928 para Checagem e 0,967 para Anotações, resultando em um CVR-S global de 0,940. Esses resultados indicam que os itens do *checklist* atendem adequadamente aos objetivos propostos, confirmando seu potencial para promover uma comunicação efetiva entre os profissionais de enfermagem da UTIN, público-alvo a que o *checklist* se destina.

A partir das sugestões dos especialistas, foram identificadas lacunas no conteúdo inicial do *checklist*, o que levou à inclusão de 32 novos itens, redistribuídos proporcionalmente entre as três categorias, conforme a lógica de integração entre ações, checagem e registros. Especificamente, foram adicionados 11 itens à categoria Procedimentos, cinco à categoria Checagem e 16 à categoria Anotações, respeitando a interdependência entre as categorias - todo procedimento executado deve ser, obrigatoriamente, checado e documentado.

Adicionalmente, avaliou-se o atributo Amplitude, correspondente à capacidade de o *checklist* de cobrir, de forma abrangente, o fenômeno em estudo. De acordo com 18 dos 19 especialistas (94,73%), o *checklist* apresentou amplitude adequada para avaliar a comunicação entre os profissionais de enfermagem no contexto da UTIN. Todos os itens foram considerados objetivos, claros, simples e relevantes.

Em virtude das alterações realizadas e da inclusão de novos itens, foi conduzida uma segunda rodada de avaliação com os especialistas, a fim de validar o conteúdo acrescido. O item “Encaminhamento inter-hospitalar (exames/cirurgias) realizado” obteve 90% de concordância, inferior ao valor crítico de CVR = 1,000. No entanto, foi mantido no *checklist* por apresentar CVR-I e CVR-S iguais a 1,000 na categoria Anotações, assegurando sua relevância no contexto do registro de informações assistenciais.

Todos os itens da categoria Checagem atingiram 100% de concordância no atributo Relevância, sendo assim considerados válidos. Quanto à categoria Anotações, embora a maioria dos itens tenha apresentado concordância superior a 80%, o item “Medidas para prevenção de quedas registradas” não atingiu o valor crítico para o atributo Relevância e, por isso, foi excluído do *checklist*.

Após a realização das duas rodadas de avaliação e consolidação das análises estatísticas (CVR-I e CVR-S), o *checklist* (versão final) foi composto por 84 itens, distribuídos em 31 itens na categoria Procedimentos, 23 em Checagem e 30 em Anotações de Enfermagem. O *checklist* obteve um CVR médio (global) de 0,948 e encontra-se disponível em Suplemento 1.

## DISCUSSÃO

A construção e validação de *checklists* voltados à segurança do paciente em contextos de alta complexidade, como as UTIN, constituem iniciativas fundamentais para qualificar a prática assistencial. Neste estudo, o desenvolvimento de um *checklist* para apoiar a comunicação efetiva entre os profissionais de enfermagem representa um avanço significativo na sistematização de estratégias de segurança direcionadas ao cuidado neonatal. Os resultados demonstram que *o checklist* elaborado apresenta atributos de representatividade, relevância e clareza nos domínios de conteúdo, os quais constituem critérios amplamente utilizados na avaliação da validade de conteúdo e considerados fundamentais nesse processo^(29)^.

A construção inicial do *checklist* baseou-se em diretrizes visuais e estruturais para favorecer a legibilidade, a compreensão e a aplicabilidade do *checklist*. A escolha por um *layout* simples, estruturado e sistematizado, aliado à categorização lógica dos itens em Procedimentos, Checagem e Anotações, pretende facilitar a organização dos dados clínicos e reforçar a interdependência entre ações assistenciais e registros, aspecto amplamente defendido como essencial à continuidade do cuidado^(30)^.

A primeira etapa deste estudo envolveu uma RIL^(21)^, consultas aos instrumentos de referência em segurança do paciente^(22,23)^ e a opinião de enfermeiros, com o objetivo de contemplar os diversos aspectos avaliativos necessários à construção de um *checklist* confiável e válido. Esta etapa fundamentou uma definição conceitual clara e racional do construto a ser validado, baseada no uso pretendido, anterior à formulação conceitual. A validade do construto dependeu, portanto, de uma definição teórica sólida, e não exclusivamente de testes psicométricos^(31)^.

A construção do *checklist* por meio de uma abordagem participativa, consultiva e integrada ao fluxo de trabalho contribuiu para priorizar o contexto real em que a pesquisa foi conduzida, a qual foi fundamental na fase de planejamento, e será crucial nas fases de implementação e análise a serem realizadas futuramente. Desse modo, buscou-se usar uma abordagem pragmática para acelerar a tradução de evidências em prática^(32)^.

Nesse contexto, a RIL desempenhou papel norteador ao identificar lacunas no conhecimento que fundamentaram o conteúdo do *checklist* ^(33)^ e a participação ativa de enfermeiras da UTIN atuantes no hospital regional na construção da segunda versão do *checklist* adequou o conteúdo à prática cotidiana. A abordagem participativa, que envolveu sugestões em diferentes momentos e a testagem prática do *checklist*, conferiu robustez ao processo e alinhamento às necessidades da equipe, conforme evidenciado em estudos similares^(16,34)^.

Na etapa de validação, a diversidade geográfica e acadêmica dos especialistas agregou valor à avaliação de conteúdo, promovendo uma análise mais abrangente do *checklist* sob diferentes perspectivas. A distribuição dos especialistas por quatro regiões brasileiras e sete estados, embora com predominância do Nordeste, contribuiu para a heterogeneidade da amostra, aspecto essencial à validação transcultural e à aplicabilidade em diferentes contextos de saúde^(35,36)^. Seguiu-se, portanto, a premissa de que especialistas deviam possuir a *expertise* de conteúdo, representatividade e embasamento teórico suficientes para fornecer uma avaliação abrangente do *checklist* ^(37)^.

Embora a maioria dos itens tenha apresentado valores de CVR-I e CVR-S satisfatórios, acima da linha de base, conforme parâmetros da literatura^(28,29)^, alguns itens ficaram abaixo do ponto de corte nos atributos Clareza e Relevância. A decisão de mantê-los para reavaliação, com base nas contribuições qualitativas dos especialistas e em valores ainda dentro da faixa aceitável^(38)^, reflete o compromisso com a pertinência clínica e a coerência com os objetivos do *checklist*. Tal estratégia está em conformidade com abordagens que priorizam a usabilidade em contextos reais^(28)^.

A expansão do *checklist* com 32 novos itens redistribuídos nas categorias originais demonstra sua capacidade de evoluir com base em evidências práticas e teóricas. Este processo respondeu à necessidade de evitar a sub-representação de conteúdo, garantindo que todos os elementos essenciais do construto fossem contemplados. Para isso, foi imprescindível aplicar criteriosamente a teoria e o julgamento de especialistas, assegurando a coerência entre os elementos incluídos, os objetivos do estudo e as definições operacionais adotadas^(29)^.

Na segunda rodada de validação, embora o item “Encaminhamento inter-hospitalar (exames/cirurgias) realizado” não tenha atingido o valor crítico de CVR para Relevância, ele foi mantido com base em sua importância assistencial e em outros parâmetros estatísticos. Por outro lado, o item “Medidas para prevenção de quedas registradas” foi excluído por não atingir os requisitos mínimos, o que demonstra o rigor metodológico adotado e o compromisso com a excelência técnica do *checklist*. A exclusão de conteúdos irrelevantes é fundamental para evitar ameaças à representatividade e preservar a validade de conteúdo do *checklist* ^(29)^.

A versão final do *checklist*, composta por 84 itens (31 Procedimentos, 23 Checagens e 30 Anotações), obteve um CVR global de 0,948, indicando um nível quase perfeito de concordância entre os especialistas. A análise do atributo Amplitude mostrou que 94,73% dos avaliadores consideraram o conteúdo abrangente e representativo da complexidade da comunicação em UTIN, corroborando a pertinência dos itens selecionados^(38)^. A validação do *checklist* constituiu etapa essencial, permitindo a avaliação criteriosa e o aperfeiçoamento do *checklist*, culminando na sua terceira e última versão. Considerada adequada pelos especialistas, esta versão foi recomendada para uso em UTIN, consolidando-se como instrumento eficaz para o fortalecimento das competências comunicacionais da equipe de enfermagem^(37)^.

Apesar disso, é preciso considerar a necessidade de aplicabilidade do *checklist* desenvolvido na prática real. Propõe-se uma intervenção multifacetada para possibilitar a adesão e impactar os desfechos clínicos^(39)^. A implementação inicial deve incluir preparação, envolvendo a liderança da unidade, garantindo a disponibilidade dos recursos necessários antes da implementação e treinamento sobre o uso do *checklist*, que pode ser complementado com treinamento técnico adicional. As abordagens de suporte contínuo também podem incluir mentoria regular no local, mensagens de texto em grupo para solução de problemas, treinamento de atualização e reuniões regulares de revisão de segurança ou qualidade^(40)^.

Ademais, é preciso atuar para que o uso do *checklist* não seja percebido pela equipe de enfermagem apenas como acréscimo de novas práticas à sua rotina diária para aumentar a carga de trabalho, mas como uma estratégia que irá diminuir a ocorrência de eventos adversos. Para tal, as instituições de saúde devem adotar a cultura de segurança e capacitação dos profissionais para utilizá-lo^(41)^.

### Limitações do estudo

Como limitação do presente estudo, aponta-se o fato de o *checklist* ainda não ter sido testado em ambiente clínico real, etapa que se encontra em desenvolvimento por parte da equipe de pesquisa. Ainda assim, a validação do conteúdo representa um avanço significativo para a enfermagem e para a área da saúde como um todo, ampliando as possibilidades de incorporação de estratégias voltadas à promoção da segurança do paciente em contextos neonatais.

### Contribuições para a área da Enfermagem

O *checklist* desenvolvido neste estudo mostra-se um instrumento promissor para qualificar a transição de cuidados entre turnos em UTIN, promovendo a continuidade assistencial e a padronização dos processos. Sua aplicação pode melhorar a comunicação entre profissionais^(16)^, reduzir falhas e fortalecer práticas seguras, conforme evidenciado por estudos que destacam a eficácia dos *checklists* na melhoria de desfechos clínicos^(42)^, especialmente em contextos pediátricos e de alta complexidade^(18,43)^.

## CONCLUSÕES

A construção do *checklist* para apoiar a comunicação efetiva dos cuidados de enfermagem na unidade de terapia intensiva neonatal fundamentou-se em três dimensões - teórica, operacional e empírica - além de se apoiar em evidências científicas relacionadas à segurança do paciente. O processo de construção envolveu enfermeiros diretamente atuantes no contexto assistencial, enquanto a validação de conteúdo foi realizada por um grupo de especialistas, que consideraram o *checklist* adequado e recomendaram sua utilização após a incorporação dos ajustes sugeridos. Assim, a adoção deste *checklist*, específico para o ambiente neonatal, configura-se como uma estratégia relevante para assegurar uma passagem de plantão mais segura e eficiente entre os profissionais de enfermagem. Vislumbra-se que o *checklist* elaborado possibilita o desenvolvimento de uma prática da enfermagem que contribuirá para a melhoria contínua da qualidade do cuidado.

## Data Availability

Os dados de pesquisa estão disponíveis no corpo do artigo.
